# The p110*α* and p110*β* Isoforms of Class I Phosphatidylinositol 3-Kinase Are Involved in Toll-Like Receptor 5 Signaling in Epithelial Cells

**DOI:** 10.1155/2010/652098

**Published:** 2010-10-03

**Authors:** Sabine M. Ivison, Mohammed A. S. Khan, Nicholas R. Graham, Leila A. Shobab, Yu Yao, Arnawaz Kifayet, Laura M. Sly, Theodore S. Steiner

**Affiliations:** ^1^Division of Infectious Diseases, Department of Medicine, Vancouver Coastal Research Institute (VCRI), Faculty of Medicin, University of British Columbia, Rm D452 HP East, 2733 Heather St., Vancouver, BC, Canada V5Z 3J5; ^2^Child and Family Research Institute, B.C. Children's Hospital, Vancouver, BC, Canada V6H 3V4

## Abstract

*Background*. Bacterial flagellin triggers inflammation in mammalian cells via Toll-like receptor (TLR) 5. Release of the chemokine IL-8 in response to flagellin involves NF-*κ*B, p38 MAP kinase, and phosphatidylinositol 3-kinase (PI3K). However, PI3K has been reported to be either pro- or anti-inflammatory in different model systems. We hypothesized that this could be due to different activities of the p110*α* and *β* isoforms of PI3K. *Results*. PI3K and Akt were rapidly activated in Caco-2 colon carcinoma cells by flagellin. Using a plasmid-based shRNA delivery system and novel p110 isoform-specific inhibitors, we found that flagellin-induced IL-8 production was dependent on both p110*α* and p110*β*. However in the mouse, inhibition of p110*β* but not p110*α* reduced the increase of serum IL-6 levels induced by intraperitoneal injection of flagellin. *Conclusions*. These data demonstrate that the p110*α* and *β* isoforms of class IA PI3K are both required for the proinflammatory response to flagellin.

## 1. Background

Flagellin is the major structural protein of bacterial flagella, the organelles that confer motility to a wide variety of bacterial species. Flagellin is also a potent trigger of innate immune responses in eukaryotic cells from plants [1] and vertebrates (reviewed in [2]). Most if not all of the responses to flagellin in vertebrate epithelial cells are mediated by Toll-like receptor (TLR) 5 [3–7]. In general, activation of TLRs by microbial products can lead to the induction of inflammatory responses via a MyD88-dependent or—independent pathways, leading to I-*κ*B degradation and ultimately NF-*κ*B and/or IRF-3 activation [8]. However, divergence and modulation of TLR signalling with the receptor/ligand pair is an important component of the innate immune response. This divergence implicates the involvement of signalling components other than NF-*κ*B. We and others previously reported that flagellin-induced production of the neutrophil chemokine interleukin (IL)-8 requires the activity of p38 MAP kinase, and that inhibition of NF-*κ*B with the I-*κ*B degradation inhibitor Bay11-7082 results in only slight inhibition of this TLR5-mediated response [9–11]. Thus, IL-8 production in response to flagellin challenge depends significantly on NF-*κ*B-independent pathways.

Phosphatidylinositol 3-kinase (PI3K) has been implicated in NF-*κ*B-dependent and –independent inflammatory signaling following TLR ligation. Class IA PI3K consists of a p85 regulatory subunit and one of several p110 catalytic subunits (*α*, *β*, or *δ*), whose primary activity is the addition of a phosphate to the 3' position of the phosphatidylinositol-4,5-diphosphate ring. The class IB PI3K, comprised of p101 regulatory and p110*γ* catalytic subunits, has a similar activity. The resulting product of both enzymes, PI-3,4,5-P_3_, activates downstream signaling through several kinases, most notably Akt and 3-phosphoinositide-dependent kinase (PDK) 1, leading to cellular responses that vary with the cell type and stimulus studied [12]. A role for PI3K in TLR5 signaling was shown in two previous studies. The first of these, by Yu et al. [9], found that inhibition of PI3K with the broadly-reactive PI3K inhibitors wortmannin (WM) or LY294002 (LY29) increased IL-6 and IL-8 production in response to flagellin in T84 cells, suggesting that PI3K is inhibitory in the flagellin-mediated signaling of intestinal epithelial cells (IECs). Moreover, they found that systemic cytokine release from PI3K p85*α* −/− mice in response to intraperitoneal injections of flagellin was significantly higher than in heterozygous littermates. WM increased MAPK activation but not I-*κ*B degradation in response to flagellin. The second publication, by Rhee et al. [13], demonstrated Akt activation in murine colon and nontransformed human colonic cells (NCM460) treated with flagellin. In contrast to Yu et al, they found that inhibition of PI3K using dominant-negative p85 or Akt, or LY29, reduced IL-8 production in response to flagellin, indicating that PI3K augments flagellin-mediated inflammatory responses in IECs. 

To help reconcile these discrepant results, we first confirmed that flagellin stimulates PI3K enzymatic activity in Caco-2 cells, and then assessed the impact of PI3K inhibition on flagellin-mediated inflammatory signaling by directly inhibiting the two class 1A PI3K isoforms expressed in IECs, p110*α*, and p110*β* [14], using both RNA interference and novel, isoform-specific pharmacological inhibitors. We found that inhibition of p110*α* or *β* significantly decreased flagellin-induced IL-8 release, although inhibition of these isoforms produced distinct effects on MAPK activation and IL-8 mRNA concentrations in human IECs as well as in an *in vivo* mouse model of flagellin inflammatory responses. The findings suggest that the PI3K pathway has a net proinflammatory effect in TLR5 signaling, and that the discrepant results reported in the literature could be due to differences in p110 isoform activities.

## 2. Methods

### 2.1. Reagents

Culture media and supplements were purchased from Sigma (St. Louis) except where otherwise indicated. Antibodies included mouse anti-PK (Serotec; Oxford, UK); mouse anti-PI3K p85, rabbit anti-pSer_473_Akt, and mouse 4G10 antiphosphotyrosine (Upstate; Charlottesville, VA); rabbit antiphospho-p38 T_180/_Y_182_, total p38, phospho-p44/42 T_202/_Y_204_, total p44/42 (Cell Signaling; Beverly, MA); mouse anti-GAPDH (RDI; Flanders, NJ); rabbit anti-I-*κ*B*α* C-15 and goat anti-actin (Santa Cruz Biotechnology; Santa Cruz, CA); HRP goat anti-mouse (Cedar Lane; Hornby, ON); HRP mouse anti-rabbit IgG (Sigma). LY294002, Bay 11-7085, and LY303511 were purchased from Calbiochem (San Diego). Isoform-specific PI3K inhibitors for *in vitro* studies were provided by Kevan Shokat (University of California, San Francisco) [7, 15]. TGX-221 and PI-103 used in mouse experiments were purchased from Chemdea (Ridgewood, NJ) Recombinant LPS-free *E. coli* H18 flagellin was produced as previously described in [16]. The human IL-8 and mouse IL-6 ELISAs were from R&D Systems (Minneapolis, MN). Flagellin was used at saturating concentrations (500–1000 *μ*g/ml) for proximal signaling events in Caco-2 cells, and at 100 ng/ml in experiments involving PI3K inhibitors or shRNA as this dose is the threshold dose for maximal IL-8 production.

### 2.2. Cell Culture

 Caco-2 cells were obtained from the American Type Culture Collection (ATCC, Rockville, MD) and grown in DMEM with 4.5 g/L D-glucose, 1x nonessential amino acids, 2 mM glutamine, penicillin (100 U/ml), and streptomycin (100 *μ*g/ml) with 10% fetal bovine serum (FBS; Hyclone, Logan, UT). Caco-2 cells were seeded at a density of 10^6^/ml and used for experiments 5–14 days after becoming confluent. HEK 293T cells were from ATCC and were grown in DMEM with 10% heat-inactivated FBS, penicillin, streptomycin, and nonessential amino acids. T84 cells from ATCC were grown in DMEM/F12 with 15 mM HEPES, pen/strep, and 5% FBS. They were seeded at 5 × 10^5^ per well and used 3–4 days after seeding.

### 2.3. Western Blotting and Immunoprecipitation

Flagellin treatment of cells cultured in 6- or 12-well plates for various time points was followed by two washes with ice-cold phosphate-buffered saline (PBS). Treated cells were then lysed in 250–500 *μ*l of lysis buffer (20 mM Tris pH 7.5, 150 mM NaCl, 1 mM EDTA, 1 mM EGTA, 1% NP-40, 2.5 mM sodium pyrophosphate, 1 mM *β*-glycerophosphate, 1 mM Na_3_VO_4_, 1 *μ*g/ml leupeptin, and 1 mM PMSF) and equal volumes of protein lysate from each sample analyzed by Western blotting. For immunoprecipitation of PI3K, 5 *μ*g of anti-p85 was added to 500 *μ*g of cell lysate and incubated at 4°C overnight. Immune complexes were bound to protein A-agarose for 1 hour, washed in lysis buffer, and analyzed by Western blot using 4G10 at 1 : 1000.

### 2.4. IL-8 Promoter/Reporter Assays

Caco-2 cells were electroporated with pEGFP and IL-8 promoter-luciferase reporter plasmids and seeded in 96-well plates as described in [16]. After 6 days, cells were stimulated and lysed in Bright-Glo reagent (Promega, Madison, WI). The ratio of luminescence to fluorescence in arbitrary units (to correct for cell number and transfection efficiency) was calculated for each sample, and the fold increase in this value compared to controls within the same experiment was defined as the fold increase in expression.

### 2.5. In Vitro Kinase Assay for PI3K

Caco-2 cells were grown at least 5 days postconfluence in 6-well plates. They were serum starved overnight, and then treated with flagellin for brief time points (1–30 minutes). Cells were rinsed with cold PBS, lysed, and immunoprecipitated with anti-p85 and protein A agarose. During the second wash step, 20% of the total volume of beads from each tube was removed and analyzed by Western blot for total amount of p85 (IP control). Remaining immunoprecipitates were subjected to in vitro kinase assay as described in [17]. Kinase autoradiographs and p85 Western blots were scanned and analyzed by densitometry, and the ratio of these two values calculated for each sample.

### 2.6. Electrophoretic Mobility Shift Assay for NF-*κ*B

Caco-2 cells seeded in 6-well plates were used at least 7 days after plating. Cells were fed with 1 ml fresh growth medium and incubated with signaling inhibitors for 60 minutes followed by flagellin or IL-1*β* for 30–60 minutes. Nuclear extracts were incubated with ^32^P-labeled NF-*κ*B-binding oligonucleotide and analyzed by EMSA as described in [18].

### 2.7. Quantitative RT-PCR

Caco-2 cells grown to differentiation in 24-well plates were pretreated with pharmacologic inhibitors for 30 minutes and then stimulated for 60 minutes. Cells were lysed and cytoplasmic RNA isolated (Rneasy mini, Qiagen; Mississauga, ON). Equal amounts of RNA from each sample were reversely transcribed using Superscript II (Invitrogen), and equal volumes from each reaction subjected to real-time PCR using Sybr Green (Applied Biosystems; Foster City, CA) for fluorescent detection. Fold changes in IL-8 mRNA were standardized to GAPDH standards for each sample and calculated as described in [18].

### 2.8. Construction and Analysis of shRNA Mediated Knockdown of p110 Subunits

Target sequences for shRNA-mediated knockdowns of p110*α* and p110*β* were as follows: PIK3CA (Acc. NM_006218), 224-244, and PIK3B (Acc. NM_006219) 1140-1161. Knockdown vectors were constructed by cloning the following oligonucleotides into the BglII and HindIII sites of pSUPER (OligoEngine): p110-*α*1-ATGTTTACTACCAAATGGA (226-244); p110-*α*2-GGTGGAATGAATGGCTGAA (1302-1320); p110-*β*1-TGGAATGAACCACTGGAAT (1141-1159); p110-*β*2-CTGTCACTTGTGGGATTGT (481-499). HEK-293T cells were seeded at 10^5^/well in 24-well plates and transfected 16–24 hours later with 50 ng of TLR5 and 500 ng of pSuper or derived knockdown vector plus 1.25 *μ*l of Lipofectamine 2000 (Invitrogen) per well, according to the manufacturer's instructions. After an additional 24 hours the medium was changed, and cells were stimulated with 500 ng/ml of flagellin 48 hours after transfection. Supernatant was harvested for IL-8 determination after 6 hours, and mRNA was quantified by QPCR as described above using the following oligonucleotides: p110*α*5′-ACGTGTGCCATTTGTTTTGAC and 5′-TTATGAAGAGATTGGCATGCTG; p110*β*: 5′-AAAAAACTGGCCAGCTCT and 5′-TAATGCAAGAGAGTCCTT. Knockdown ratios were expressed as a fold decrease in mRNA expression compared to cells expressing empty pSUPER vector, using the calculation method described above for Caco-2 cells.

### 2.9. Mouse Injections

6-12-week-old female C57Bl/6 mice bred in our animal care facility were cared for in accordance with the guidelines of the UBC Animal Care and Use Committee in accordance with Canadian regulations. Mice were injected intraperitoneally with inhibitors or DMSO vehicle as described in Results, followed 30 minutes later by 10 *μ*g of flagellin in 100 *μ*l of sterile phosphate-buffered saline (PBS). Blood was taken via the saphenous vein prior to injection and 90 minutes and 3 hours after flagellin injection. At 6.5–7 hours after injection, mice were euthanized by CO_2_ asphyxiation and blood taken by cardiac puncture. Sera were stored at −80°C until testing for IL-6 by ELISA.

### 2.10. Statistical Analysis

Statistics were performed on original data using the VassarStats statistical computation website (http://vassun.vassar.edu/~lowry/VassarStats.htm). Except where noted, multiple groups were analyzed by ANOVA to verify the presence of significant differences; this was followed by testing of individual pairs by t-test or Tukey HSD.

## 3. Results

### 3.1. Flagellin Causes Enzymatic PI3K Activation in Caco-2 Cells

**Figure 1:**
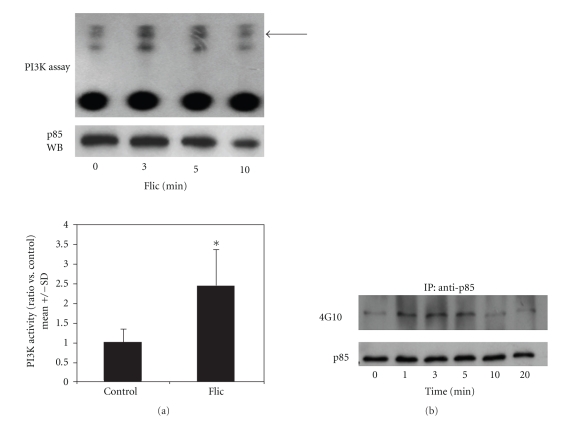
Activation of PI3K in Caco-2 cells by *E. coli* flagellin. (a) *In vitro* kinase assay. Caco-2 cells treated with flagellin (FliC) 1 *μ*g/ml for the indicated times were lysed and immunoprecipitated with anti-p85. An aliquot of each immunoprecipitate was analyzed by Western blot for p85, and the remainder was reacted with phosphatidylinositols and *γ*-^32^P-ATP and separated by thin-layer chromatography. The PIP3 band is indicated by the arrow. Densitometry performed on autoradiograms from three independent experiments is shown below. *P < .05, FliC versus control (*t*-test). (b) Tyrosine phosphorylation of PI3K. Caco-2 cells treated with 500 ng/ml flagellin for the indicated times were lysed and immunoprecipitated with anti-p85. Precipitates were separated by SDS-PAGE and Western blots performed using 4G10 antiphosphotyrosine. Blots were stripped and reprobed with anti-p85 to confirm equal efficiency of immunoprecipitation. Results are representative of two separate experiments.

Activity of PI3K in cell lysates was measured by in vitro kinase assay. As shown in Figure 1(a), Caco-2 cells possessed measurable basal PI3K activity, consistent with their transformed nature and reduced intrinsic PTEN activity [19]. Within 5 minutes of stimulation with flagellin, a rapid and transient increase in PI3K activity was measured. This activity returned to baseline after 30 minutes (not shown). As an additional test for PI3K activation, Caco-2 lysates were immunoprecipitated with anti-PI3K p85 followed by Western blot using antiphosphotyrosine (4G10). As shown in Figure 1(b), flagellin-treated cells had a time-dependent increase in signal versus controls, indicating tyrosine phosphorylation of PI3K, which is associated with PI3K enzymatic activation [20].

### 3.2. Flagellin-Induced NF-*κ*B Activity Is Not PI3K Dependent

**Figure 2:**
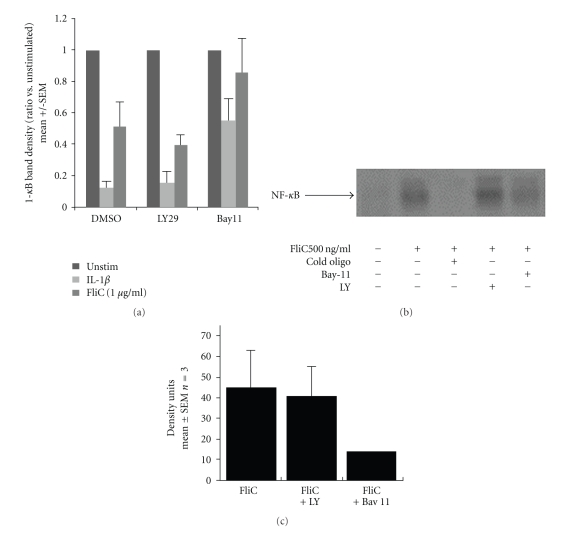
Flagellin-induced I-*κ*B degradation and NF-*κ*B activation in Caco-2 cells are PI3K independent. (a) I-*κ*B*α* degradation in Caco-2 cells treated with IL-1*β* (10 ng/ml) or flagellin (FliC, 1000 ng/ml or 500 ng/ml) was not inhibited by LY29 (30 *μ*M) but was inhibited by Bay 11-7082 (20 *μ*M). Caco-2 cell lysates were analyzed by Western blot for anti-I*κ*B*α* and anti-GAPDH and the band density measured. Density of I-*κ*B bands was divided by GAPDH to normalize for protein loading and transfer, and the ratio was taken versus unstimulated cells in each experiment to calculate the amount of I-*κ*B*α* degradation. Results were pooled from four separate experiments. *P* < .01 versus DMSO by ANOVA, DMSO + FliC versus Bay11 + FliC. (b) NF-*κ*B activation in Caco-2 cells measured by electrophoretic mobility shift assay, with the shifted NF-*κ*B band indicated by the arrow. (c) Densitometry measurements of NF-*κ*B-bound probe bands, pooled from 3 independent experiments.

 We next analyzed I-*κ*B degradation and NF-*κ*B localization and DNA binding activity in response to flagellin. As shown in Figure 2(a), flagellin-induced degradation of I-*κ*B in Caco-2 cells was not significantly affected by LY29, but was reduced by Bay11-7082. We next examined NF-*κ*B nuclear localization and DNA binding activity by EMSA. As shown in Figures 2(b) and 2(c), flagellin caused a large increase in NF-*κ*B activity in Caco-2 cells that was not reduced by LY29.

### 3.3. PI3K Inhibition Reduces IL-8 Promoter Activity, mRNA Production, and Secretion in Caco-2 Cells

**Figure 3:**
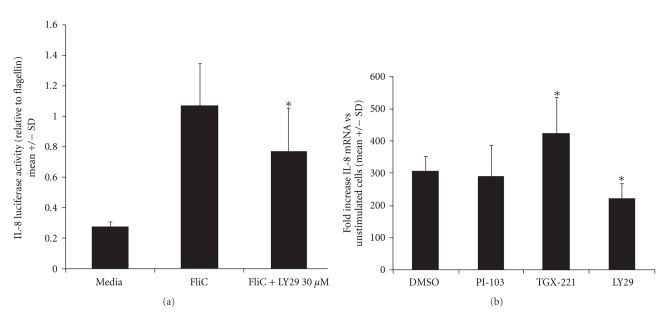
PI3K inhibition reduces flagellin-stimulated IL-8 promoter activation and mRNA production. (a) Caco-2 cells were electroporated with and IL-8 promoter/luciferase reporter plasmid and pEGFP as a transfection marker and grown as described in Methods. Cells were pretreated with inhibitors at the concentrations shown (or an equivalent volume of DMSO vehicle) followed 30 minutes later by flagellin 100 ng/ml. After 6 hours, cells were harvested for luciferase assay and fluorescence determination. The RLU/RFU value was calculated for each well, and these data normalized to the wells stimulated with DMSO + flagellin in each individual experiment. Data shown are compiled from at least 3 experiments for each inhibitor. *P < .01 (*t*-test). (b) IL-8 mRNA measurements by quantitative RT-PCR compiled from at least 3 experiments. Cytoplasmic RNA isolated from cells treated as shown was reverse transcribed. cDNA was amplified using actin and IL-8 primers with real-time fluorescent quantitation. Fold increases in mRNA were calculated as described in Methods. Results are expressed as the ratio compared to cells treated with flagellin plus DMSO vehicle. **P < .05 versus flagellin plus DMSO (*t*-test).

Yu et al. [9] showed that LY29 increased IL-8 production in T84 transformed human colonocytes. This stands in contrast to Rhee et al. [13], who found that LY29 inhibited flagellin responses in NCM460 nontransformed human colonocytes. To reconcile this discrepancy, we measured IL-8 production in Caco-2 cells. As shown in Figure 3(a), Caco-2 cells transiently transfected with an IL-8 promoter/luciferase reporter construct that had approximately a 4-fold increase in luciferase expression after treatment with flagellin. Pretreatment of cells with 30 *μ*M LY29 reduced flagellin-induced luciferase expression by about one-fourth (0.76 ± 0.29; *P* < .05). LY29 also inhibited IL-1*β*-induced luciferase expression (0.54 ± 0.023; *P* < .001, *t*-test; not shown). 

The effect of PI3K inhibition on IL-8 mRNA production was measured by quantitative real-time RT-PCR. As shown in Figure 3(b), flagellin caused a marked increase in IL-8 mRNA at 1 hour compared to untreated cells. LY29 reduced flagellin-stimulated IL-8 mRNA amounts by about one-third compared to DMSO vehicle (*P* < .05). As LY29 has been reported to affect kinases other than PI3K, such as the mammalian target of rapamycin (mTOR) and casein kinase 2 (CK2), we tested an analogue, LY303511 (LY30), which lacks PI3K inhibitory activity but does inhibit mTor and CK2 [21–23]. The inactive analog, LY30, did not significantly reduce IL-8 mRNA expression (not shown). In contrast, two isoform-specific PI3K inhibitors (described below) showed disparate effects, with TGX-221 (p110*β*) causing an increase in IL-8 mRNA, and PI-103 (p110*α*) producing no effect (Figure 3(b)).

**Figure 4:**
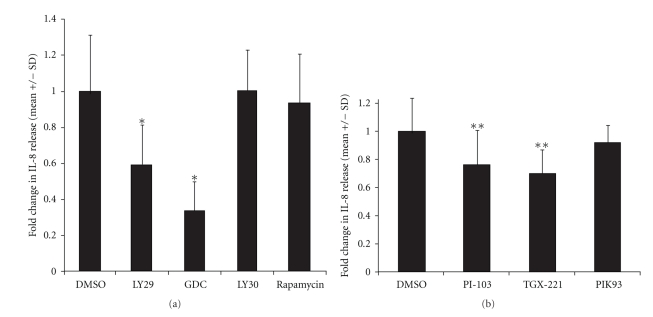
PI3K inhibition reduces IL-8 production after flagellin stimulation in Caco-2 cells. Caco-2 cells seeded as described in Methods were treated with the inhibitors shown (all at 10 *μ*M, except for 30 *μ*M LY29 and LY30) or an equivalent dose of DMSO vehicle in complete culture media. Thirty minutes later, flagellin was added to a final concentration of 100 ng/ml, and cell supernatants harvested 3 hours later. IL-8 was measured by ELISA, and the values in each experiment were normalized to the flagellin + DMSO wells. (a) Effects of broadly reactive PI3K inhibitors. (b) Effects of isoform-specific inhibitors. *P < .001 versus DMSO; **P < .01 versus DMSO (*t*-test). Results compiled from N of at least 10 for each inhibitor.

We next measured the effects of PI3K inhibitors on IL-8 release from flagellin-stimulated Caco-2 cells. For these experiments we used LY29 as well as a newly-available, broadly-reactive PI3K inhibitor, GDC0941. Pretreatment of cells with LY29 or GDC0941 significantly inhibited flagellin-induced IL-8 release (Figure 4(a)). We also tested effects of LY30, rapamycin (the prototype mTOR inhibitor), and pp242 (a novel mTor inhibitor). None of these inhibitors significantly inhibited flagellin-induced IL-8 release from Caco-2 cells, suggesting that the effects seen with LY29 and GDC0941 were in fact due to PI3K inhibition rather than off-target effects on mTOR or CK2. (Figure 4(a)). Absolute values of released IL-8 in the DMSO + flagellin treated cells ranged from 400–800 pg/ml.

### 3.4. p110*α* and p110*β* Are Both Required for Flagellin-Induced Signaling in Caco-2 Cells

To determine whether flagellin-induced inflammation requires a particular isoform of class Ia PI3K, we employed a panel of novel chemical agents with specificity for different p110 catalytic subunits. A list of these and standard agents is found in Table 1. In addition to LY29, TGX-221, and PI-103 significantly inhibited IL-8 release, despite their disparate effects on IL-8 mRNA (Figure 4(b)). Both compounds also inhibited CCL20 release from Caco-2 cells, as did LY29 (see Figure 4(c) in Supplementary Material available online at doi: 10.1155/2010/652098). In contrast to Caco-2 cells, T84 cells showed no significant change in flagellin-induced IL-8 production with LY29, TGX-221, or PI-103, and had a significant increase in IL-8 with WM (4.8 ± 1.4-fold increase; *P* < .001, *t*-test, see Figure 4d in Supplementary Material). These results support those of Yu et al. [9], and suggest that PI3K signaling in IECs varies between cell types.

**Figure 5:**
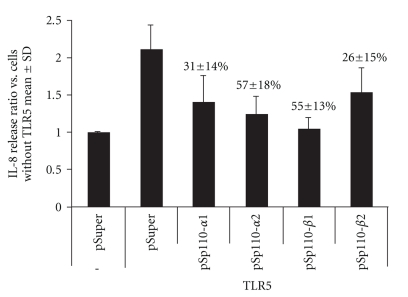
Effect of p110*α*- and p110*β*-targeted shRNA on IL-8 production in TLR5-transfected, flagellin-stimulated HEK293T cells. Cells were transfected with TLR5 and a pSuper derivative or with pSuper alone and then stimulated with 500 ng/ml flagellin for 6 hours. IL-8 concentrations were determined by ELISA. pSp110-*α*1 and -*α*2 target 2 separate sites of p110*α*, while pSp110-*β*1 and -*β*2 target two separate sites of p110*β*. Results show fold increase versus baseline (IL-8 amounts measured in flagellin stimulated cells which were not transfected with TLR5). The percentages above the columns denote the amount of knockdown of either p110*α* or p110*β* mRNA as determined by quantitative RT-PCR; results were compiled from at least 7 samples from a minimum of 3 separate experiments. All four knockdowns significantly reduced IL-8 production (*P* < .001, ANOVA).

As the specificity of pharmacological inhibitors is imperfect, we sought to confirm these results, using RNA interference. shRNA constructs were cloned into the pSuper vector, using sequences reported in the literature to be effective at knocking down the class IA PI3K catalytic isoforms p110*α* and p110*β*, both of which are known to be expressed in Caco-2 cells. Because Caco-2 cells have relatively low transfection rates, we looked at IL-8 secretion in HEK 293T cells, which are highly transfectable and produce IL-8 in response to flagellin when transfected to overexpress TLR5 (they are minimally responsive to flagellin otherwise). As with Caco-2 cells, LY29 decreased flagellin-mediated IL-8 release from HEK 293T cells (37.3 ± 4.1% inhibition with LY29; not shown). Multiple sequences were selected from each p110 isoform and cloned into pSuper to generate a panel of shRNA-expressing plasmids. Knockdown of specific p110 mRNA was confirmed by QPCR. The different knockdown constructs reduced IL-8 secretion in flagellin-stimulated, TLR5 transfected HEK cells in proportion with their degree of knockdown as determined by QPCR (Figure 5). All constructs shown in Figure 5 significantly reduced IL-8 secretion in comparison to pSuper transfected cells (*P* < .01 by ANOVA followed by *t*-test). As an additional control, we found that two knockdown constructs for p110*γ* failed to inhibit IL-8 production (not shown). The absolute concentration of IL-8 produced in TLR5-expressing cells carrying empty pSuper was 182.3 +/− 96 pg/ml (*N* = 4).

### 3.5. PI3K Inhibition Alters p38 and ERK Activation in Response to Flagellin

**Figure 6:**
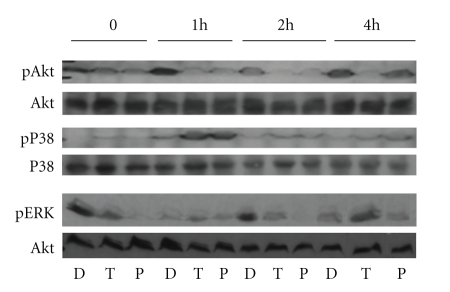
Effects of p110 inhibitors on flagellin-induced Akt, p38, and ERK activation. Caco-2 cells were serum-starved overnight, and then given serum-containing media with 10 *μ*M PI-103 (P) or TGX-221 (T) or an equivalent volume of DMSO vehicle (D). After 30 minutes, flagellin was added to a final concentration of 100 ng/ml, and cells were harvested after 1, 2, or 4 hours. Total cell lysates were separated by SDS-PAGE and tested for pSer473-Akt, phospho-p38, and phospho-ERK by Western blot. Blots were stripped and probed with total Akt and total p38 antibodies as loading controls. Results shown are typical of three independent experiments.

To determine the mechanisms by which PI3K inhibition reduced flagellin responses, we looked at the effects of PI3K inhibition on Akt and MAPK activation. It has been previously reported that flagellin causes phosphorylation of Akt, p38, and Erk kinases; moreover, Yu et al reported that PI3K inhibition led to prolonged activation of these kinases in T84 cells [9]. Both TGX-221 and PI-103 inhibited flagellin-induced Akt phosphorylation at Ser473, suggesting that the effects of flagellin on PI3K may be mediated by both p110*β* and p110*α* (Figure 6). Flagellin-induced Akt phosphorylation was also inhibited by LY29 (not shown). Interestingly, both compounds also caused a substantial increase in p38 phosphorylation at 1 hour, with less pronounced upregulation for as long as 4 hours after stimulation. In contrast, ERK phosphorylation followed an unexpected kinetic course, with both inhibitors reducing immediate and 2 hours phosphorylation, while TGX221, but not PI-103, caused a significant boost in late phosphorylation (4 hours after stimulation). Together, these results point to a complex interaction between the PI3K and MAPK pathways following TLR5 activation, and reveal different activities of p110*α* and p110*β* in the late regulatory pathways.

### 3.6. PI3K p110*β* Inhibition Reduces Inflammatory Responses to Flagellin In Vivo

**Figure 7:**
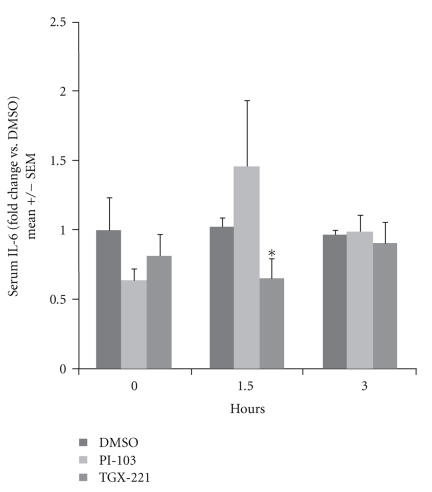
Effects of p110 inhibitors on murine responses to flagellin *in vivo*. Female C57Bl/6 mice (6–12 weeks) were injected intraperitoneally with 200 *μ*g of TGX-221 or PI-103 in 75% DMSO in PBS, or an equivalent volume of DMSO/PBS. Thirty min later, mice were injected intraperitoneally with 10 *μ*g of *E. coli* H18 flagellin in 100 *μ*l of PBS. Saphenous blood was taken after an addition 90 minutes and 3 hours. Sera were analyzed for IL-6 concentration by ELISA and results expressed as the ratio compared to flagellin plus DMSO control at each time point. Mean serum concentrations of IL-6 in the flagellin plus DMSO vehicle group were 1567 pg/ml at 90 minutes and 4915 pg/ml at 3 hours. *N* = 4 per group. *P < .05, TGX-221 versus DMSO, *t*-test.

To measure the *in vivo* relevance of PI3K isoform activation, we examined the serum IL-6 response to intraperitoneal injection of flagellin into mice. IL-6 is commonly used as a serum marker for flagellin-induced inflammatory responses in mice, which do not produce IL-8. Yu et al performed similar experiments comparing wild-type to p85*α*−/− mice and found exaggerated responses in the absence of functioning PI3K [9]. Female C57Bl/6 mice were injected with 100–300 *μ*l of TGX-221 or PI-103 in DMSO, or an equivalent volume of DMSO alone, followed 30 minutes later by 10 *μ*g of flagellin in PBS. Blood was taken at 90 minutes, 3 hours, and 7 hours following the flagellin injection, and serum IL-6 measured. Some mice also had preinjection blood taken, to establish a baseline, which in all cases was less than or equal to 125 pg/ml. All mice exhibited mildly reduced physical activity following these injections, which may be due to the known depressant effects of DMSO. No mice died or exhibited any other signs of distress such as stereotypic movements, hunched posture, ruffled fur, etc. As shown in Figure 7, mice treated with TGX-221 had a statistically significant reduction in serum IL-6 90 minutes after flagellin injection, compared to DMSO vehicle. Serum from PI-103-treated mice contained increased IL-6, although this was not statistically significant. At 3 hours, the differences were no longer evident. It should be noted that the serum half-life of PI-103 in mice is reportedly short, although tissue clearance is much slower [29]. The serum half-like of TGX-221 has not been reported, but antithrombotic activity in rats persists for at least 60–90 minutes following an intravenous bolus [16]. 

To verify the purity of our flagellin preparations, C57Bl/6 TLR5 −/− mice were injected with the same dose and preparation of flagellin and IL-6 measured. Three-hour IL-6 production was minimal in these mice compared to wild-type mice (345 ± 27.2 versus 7655 ± 378.5 pg/ml, *P* < .001, not shown). Serum IL-6 was below assay detection limits (50 pg/ml) in mice prior to flagellin treatment.

## 4. Discussion

Our results indicate that PI3K activated upon TLR5 activation by flagellin has a net proinflammatory effect in Caco-2 cells, and that this effect is not mediated through upregulation of NF-*κ*B or p38 MAPK activity. Moreover, we have shown that both the p110*α* and p110*β* isoforms contribute to this effect, although likely through different mechanisms involving ERK phosphorylation and possibly IL-8 mRNA translation, which may lead to nonredundant effects *in vivo*. 

These findings are particularly relevant because of the current controversy surrounding the role of PI3K in TLR signaling, with both pro- and anti-inflammatory effects seen in various epithelial and hematopoietic cells from mice and humans. Moreover, differences in TLR5 activity have been implicated in several human diseases, including Crohn's disease [30], systemic lupus erythematosus [31], and *Legionella* pneumonia [32]. While TLR5 is expressed on both epithelial and hematopoietic cells, these cells differ tremendously in their exposure to the natural TLR5 ligand, flagellin. Hence, it is not surprising that signaling in response to flagellin would differ between these cell types, and the involvement of downstream kinase messengers such as PI3K is one way that this difference can be achieved. 

Class IA PI3K is a proximal transducer of a wide variety of extracellular signals. Stimuli such as growth factors, G-protein coupled receptor ligands, cytokines, and vitamin D3 can recruit the p85 regulatory subunit to the plasma membrane, where the p110 catalytic subunit converts (preferentially) PI-4,5-P_2_ into PI-3,4,5-P_3_ [10]. PI-3,4,5-P_3_ binds to and regulates several protein kinases and other targets through their pleckstrin-homology domains. The most studied of these targets are phosphatidylinositol-dependent kinase 1 (PDK1), Akt (protein kinase B), tyrosine kinases, and guanosine nucleotide exchange factors [12]. Further downstream, other proteins such as I-*κ*B kinase subtypes (IKK), mTOR, protein kinase C-*ξ*, and several antiapoptotic factors can then be activated, making PI3K an important proximal element in many signal transduction pathways [33]. 

There are two distinct p85 isoforms (*α* and *β*) and four p110 isoforms (*α*, *β*, *γ*, and *δ*). The tissue distributions of these are distinct, with all four p110 isoforms expressed on hemopoietic cells but only *α*, *β*, and *γ* expressed in normal epithelial cells. Of note, p110*γ* expression is absent in colonic adenocarcinoma cells, including Caco-2 cells [15]. While all of the subunits appear to catalyze the same enzymatic reactions, there are different cellular responses associated with them, some of which may be due to unique localization, or even nonenzymatic activities. For example, p110*β* was shown to bind to Akt in the nucleus of 3T3 cells, facilitating DNA elongation during replication [34]. In contrast, p110*α* was required for cell survival in the same study. 

In TLR and IL-1*β* signaling, PI3K is believed to be recruited to the membrane by binding of p85 to a specific YXXM motif on MyD88, leading to activation of PI3K [13]. However, in the case of TLR4, binding of PI3K may result in phosphoinositide sequestration and subsequent reduced activity of the PI3K axis [35]. Overall, the net effect of PI3K activity can be either pro- or anti-inflammatory depending on the system studied. For example, inhibition of PI3K increases COX-2 upregulation in response to TNF-*α* or IL-1 in colonic epithelial cells [36] but decreases COX-2 upregulation by lauric acid in RAW macrophages [37]. Inhibition of PI3K with LY29 or WM reduces IL-8 expression in some experimental models, but not others [38–41]. Even within the same cell type, PI3K can differentially affect inflammatory gene expression. For example, LY29 reduces expression of MIP-1*α* and MIP-1*β* mRNA, but increases IL-8 mRNA in dendritic cells incubated with LPS [42]. Effects of PI3K on NF-*κ*B also vary with the system studied, and can involve either inhibition or activation of NF-*κ*B in an I-*κ*B-dependent or –independent manner [43–47]. 

One problem with many published studies of PI3K effects is that the use of pharmacologic inhibitors is inconsistent, with wortmannin (WM), LY29, or both used in different reports. While WM at concentrations below 50 nM is quite specific for PI3K, it does have activity against smooth-muscle myosin light-chain kinase (IC_50_ of 260 nM) [23]. While more stable than WM, LY29 is less potent, and inhibits CK2 and mTOR at similar concentrations at which it inhibits PI3K [21, 23]. These activities could explain different or even opposite pharmacologic effects of LY29 and WM reported in various cell systems [48–50].

Because of these issues, several more recent studies have focused on the roles of specific p110 isoforms in TLR signaling. While there are no reports examining p110 isoforms in TLR5 signaling, they have been studied in TLR4 pathways. Utsugi et al. found that p110*β*, but not *α*, positively regulated LPS-induced IL-12 production in human DCs and macrophages [51]. In contrast, in murine macrophages, Tsukamoto et al. found that shRNA-mediated inhibition of p110*α* increased Akt phosphorylation and decreased iNOS activity and IL-12 production, while p110*β* inhibition had the opposite effect on these markers [52]. They concluded that p110*β* is involved in downregulation or negative feedback of TLR signaling. While these results are consistent with the late increase in pERK that we observed following flagellin stimulation in the presence of TGX-221, in other ways our findings were quite different. For example, we found that TGX-221 and PI-103 were equally effective at reducing flagellin-induced Akt phosphorylation, and that TGX-221, but not PI-103, reduced IL-6 production in response to flagellin injection in mice.

The isoform-specific pharmacologic inhibitors of PI3K used in this study are starting to prove very useful in determining the roles of individual p110 isoforms, particularly in models where shRNA is impractical (such as primary cells or *in vivo*). For example, Sly et al. found that inhibition of p110*β* with TGX-221 increased TLR4- or TLR9-mediated IL-6 production in murine macrophages whereas p110*α*, *γ*, and *δ* inhibitors had the opposite effect [53]. The highly specific nature of these inhibitors has generated interest in their use in diverse clinical settings, including platelet inhibition and cancer treatment. However, the effects of these agents on TLR5 signaling, or indeed on IECs in any model system, have not been examined previously. 

The relevance of our findings is further increased by the existing controversy regarding the role of PI3K in TLR signaling in general, and particularly on TLR5. As mentioned above, Yu et al. and Rhee et al. found opposite effects of PI3K inhibition on TLR5 signaling (anti-inflammatory in the former and proinflammatory in the latter). An additional paper by Kato et al. reported inhibition of TLR5 signaling by WM in TLR5-transfected HEK cells, although this was not the major focus of their work [54]. We hypothesized that differential effects of p110*α* and *β* isoforms (which are the forms expressed in transformed IECs) would be a possible explanation for these discrepancies.

Our findings clearly tip the balance in favor or PI3K having a net proinflammatory activity on Caco-2 and HEK cells, in contrast to RAW cells and T84 cells. We found that inhibition of either p110*α* or p110*β* using shRNA or pharmacologic blockade led to reduced IL-8 production that was independent of the NF-*κ*B and p38 pathways. This was despite either no effect or an activating effect on IL-8 mRNA detected by RT-PCR. In fact, we found, as did Yu et al, that PI3K inhibition led to increased p38 phosphorylation in Caco-2 cells, and a strong proinflammatory effect of WM (but not LY) in T84 cells. Given that blockade of p38 and NF-*κ*B have both been shown to reduce flagellin-induced IL-8 production, this finding was somewhat surprising, and suggests that an alternative, PI3K-dependent pathway is involved in TLR5-induced IL-8 production in IECs (perhaps one involving posttranscriptional regulation). Future studies will be required to investigate proteins that may be involved in this pathway. 

An additional unexpected observation was the late increase in ERK phosphorylation after blockade of p110*β*, but not p110*α*. This finding, along with our *in vivo* results in mice, confirm that there are in fact different proinflammatory pathways utilized by p110*α* and p110*β*. To the best of our knowledge, this is the first report of the use of TGX-221 in a live animal model, and its lack of overt toxicity suggests that it could be useful in further studies whereas specific inhibition of p110*β* may be useful. One interesting observation was that the inhibition of IL-6 production was evident only at the earliest time point after stimulation. Since the serum half-life of TGX-221 is not known, this could be strictly due to pharmacokinetic effects, but it is intriguing to speculate that the late increase of pERK seen in vitro would reflect a biphasic effect of p110*β* signaling. 

In conclusion, it is apparent from our studies that the TLR5 inflammatory pathway involves activation of PI3K, and that inhibition of either p110*α* or p110*β* blocks production of IL-8 in Caco-2 IECs *in vitro* whereas only p110*β* appears to be required for IL-6 production from mice *in vivo*. Moreover, p110 inhibition reduced IL-8 production while increasing MAPK activity without altering NF-*κ*B activity, suggesting the presence of other important molecular targets of PI3K that ultimately facilitate chemokine production in response to flagellin. Future studies of these novel targets may lead to the development of novel treatments for flagellin-related diseases, including Crohn's disease and bacterial gastroenteritis.

## Supplementary Material

Figure 4c: Effect of PI3K inhibitors on flagellin-induced CCL20 secretion from Caco-2 cells. Cells were treated with TGX-221 (10 uM), PI-103 (10 uM), LY294002 (30 uM) or an equivalent volume of DMSO vehicle , followed by flagellin 100 ng/ml for 3h. Supernatants were analyzed for CCL20 concentrations by ELISA. Results shown are mean ± SD for *N* = 6. *P < 0.002; **P < 0.001 versus DMSO control.
Figure 4d: Effect of PI3K on inhibitors on flagellin-induced IL-8 secretion from T84 cells. T84 cells grown in DMEM/F12 with 5% fetal bovine serum were plated in 24-well dishes and stimulated 2d later with flagellin 100 ng/ml with or without PI3K inhibitors shown or DMSO vehicle. Supernatants were analyzed for IL-8 after 3 h. Results are expressed as a fold change in IL-8 secretion compared to flagellin plus DMSO vehicle from *N* = 4 to 6 replicates for each inhibitor. *P 0.01 versus DMSO.

## Figures and Tables

**Table 1:** Activities of pharmacologic inhibitors used in this study (IC_50_ in nM).

Enzyme	LY294002	LY303511	GDC0941	PI-103	TGX-221	PIK-93
p110*α*	500	>1000	3	8	>1000	39
p110*β*	300–973	>1000	33	88–340	7–8.5	590
p110*γ*	1000–7000	>1000	75	15	>1000	16
p110*δ*	570–1300	>1000	3	48->500	>100	120
mTor	Active	?	580	20	?	1380
other	CK2	?	Class II PI3K (0.67)			PI4KIIIb

Data compiled from [24–28].
