# Supplementary figures and images for: The p110α and p110β Isoforms of Class I Phosphatidylinositol 3-Kinase Are Involved in Toll-Like Receptor 5 Signaling in Epithelial Cells
Source: Mediators Inflamm. 2010 Oct 3;2010:652098. doi: 10.1155/2010/652098 (PMC2952946; doi:10.1155/2010/652098)

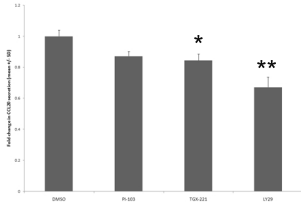

Supplement: Supplementary file 1 — Figure 4c: Effect of PI3K inhibitors on flagellin-induced CCL20 secretion from Caco-2 cells. Cells were treated with TGX-221 (10 uM), PI-103 (10 uM), LY294002 (30 uM) or an equivalent volume of DMSO vehicle , followed by flagellin 100 ng/ml for 3h. Supernatants were analyzed for CCL20 concentrations by ELISA. Results shown are mean ± SD for N = 6. *P < 0.002; **P < 0.001 versus DMSO control. Figure 4d: Effect of PI3K on inhibitors on flagellin-induced IL-8 secretion from T84 cells. T84 cells grown in DMEM/F12 with 5% fetal bovine serum were plated in 24-well dishes and stimulated 2d later with flagellin 100 ng/ml with or without PI3K inhibitors shown or DMSO vehicle. Supernatants were analyzed for IL-8 after 3 h. Results are expressed as a fold change in IL-8 secretion compared to flagellin plus DMSO vehicle from N = 4 to 6 replicates for each inhibitor. *P 0.01 versus DMSO. [file 652098.f1.pdf]

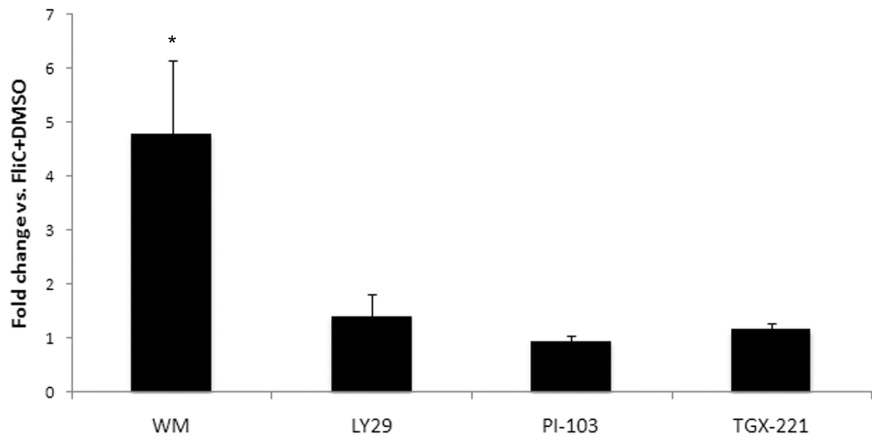

Supplement: Supplementary file 2 [file 652098.f2.pdf]
